# A Systematic Enhancer Screen Using Lentivector Transgenesis Identifies Conserved and Non-Conserved Functional Elements at the Olig1 and Olig2 Locus

**DOI:** 10.1371/journal.pone.0015741

**Published:** 2010-12-29

**Authors:** Marc Friedli, Isabelle Barde, Mélanie Arcangeli, Sonia Verp, Alexandra Quazzola, Jozsef Zakany, Nathalie Lin-Marq, Daniel Robyr, Catia Attanasio, François Spitz, Denis Duboule, Didier Trono, Stylianos E. Antonarakis

**Affiliations:** 1 Department of Genetic Medicine and Development, University of Geneva Medical School and University Hospitals of Geneva, Geneva, Switzerland; 2 School of Life Sciences, Ecole Polytechnique Fédérale de Lausanne (EPFL), Lausanne, Switzerland; 3 Department of Zoology and Animal Biology, University of Geneva, Geneva, Switzerland; 4 “Frontiers in Genetics”, National Centre for Competence in Research, Bern, Switzerland; King's College London, United Kingdom

## Abstract

Finding sequences that control expression of genes is central to understanding genome function. Previous studies have used evolutionary conservation as an indicator of regulatory potential. Here, we present a method for the unbiased *in vivo* screen of putative enhancers in large DNA regions, using the mouse as a model. We cloned a library of 142 overlapping fragments from a 200 kb-long murine BAC in a lentiviral vector expressing LacZ from a minimal promoter, and used the resulting vectors to infect fertilized murine oocytes. LacZ staining of E11 embryos obtained by first using the vectors in pools and then testing individual candidates led to the identification of 3 enhancers, only one of which shows significant evolutionary conservation. In situ hybridization and 3C/4C experiments suggest that this enhancer, which is active in the neural tube and posterior diencephalon, influences the expression of the *Olig1 and/or Olig2* genes. This work provides a new approach for the large-scale *in vivo* screening of transcriptional regulatory sequences, and further demonstrates that evolutionary conservation alone seems too limiting a criterion for the identification of enhancers.

## Introduction

The identification of sequences that control spatial, temporal and quantitative expression of genes is important to understand genome function. Other than the core promoter, several other *cis*-acting regulatory elements are required for accurate gene expression (reviewed in [1]). For instance, enhancers, by mediating expression within a specific tissue or cell type, are responsible for a subset of the total gene expression pattern. Insulators on the other hand, act as boundary elements and prevent *cis*-regulatory sequences in one gene from inappropriately interacting with adjacent loci [2]. These elements may reside in introns or up- and downstream of the transcription unit. *Cis*-regulatory domains can extend long distances outside the transcription unit; an enhancer for Sonic Hedghog for example is located one megabase away from its target gene [3]. The importance of these *cis*-acting elements has been underscored by several examples of nucleotide variation in enhancers that elicit human disorders [3], [4], [5], [6], [7].

The recent sequencing of genomes has added a pivotal tool for genome analysis in the form of comparisons and multiple alignments. These comparative genomics approaches have provided cues in the discovery of both protein-coding genes as well as potentially functional conserved non-coding elements (CNCs) [8], [9], [10], [11], [12], [13], [14]. The conclusion of these studies is that functionally relevant sequences are conserved through evolution, while the remainder of the genome evolves neutrally. Given the early availability of both the human and mouse genomes, initial efforts focused on human–mouse pair-wise comparisons; but subsequent studies frequently used more distant comparisons such as human-fish to uncover functional non-coding elements with a higher stringency [15] [16].

We previously tested the potential enhancer activity of a set of CNCs through a reporter-based assay in human cell lines, and found that only a small fraction of them scored positively [17]. However, studies using transgenic mice and more stringent evolutionary criteria demonstrated that a substantial subset of conserved non-coding sequences have transcriptional enhancer activity [13], [18], [19]. We thus developed a systematic approach to screen *in vivo* for putative enhancers in large genomic regions. Because evolutionary conservation may overlook functional elements, we further designed our method avoiding any bias towards particular sequence features. For this, we cloned a library of fragments from a mouse genomic sequence in a lentiviral vector, next to a minimal promoter-reporter cassette. We then tested the reporter gene expression by lentivector-mediated mouse transgenesis, which allowed us to generate and analyze rapidly a high number of embryos. This screen identified three enhancers in a 200 kb-long orthologous region from human chromosome 21. Interestingly, only one of these three elements, which likely controls the nearby *Olig* genes, is evolutionarily conserved.

## Results

### Generation of a lentivector-based library of candidate enhancers

**Figure 1:**
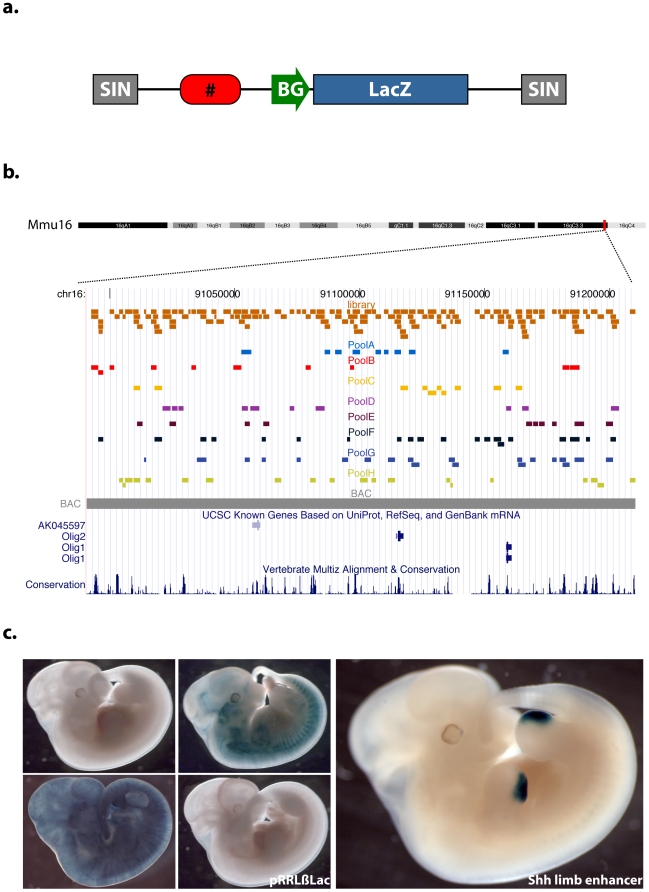
Generation of a lentiviral vector library. (A) Lentiviral vector in which each fragment of the library was cloned. SIN = self inactivating LTR, BG = beta globin promoter, LacZ = LacZ reporter. (B) UCSC genome browser view (http://genome.ucsc.edu/ [27]) of the locus screened. Orange boxes represent each clone of the library. The injected pools are shown below as coloured boxes. The corresponding segment in the human genome is included in the pilot ENCODE region ENm005 [23]. (C) Left: Embryos injected with pRRLβLac (empty vector), showing no staining, broad and unspecific β galactosidase pattern, or specific but not reproducible patterns. Right: Embryo injected with the Shh limb enhancer (positive control).

Previous studies have used evolutionary conservation as an indicator of regulatory potential, but increasing evidence suggests that this criterion alone frequently overlooks functional sequences [20]. We thus designed our study without any bias towards a particular sequence feature. We chose a mouse BAC (RP23-356P18, chr16: 90990720-91210330,Mm8) corresponding to a region of Hsa21 because it contains the *Olig1* and *Olig2* genes that are expressed specifically in the CNS [21], [22] (http://www.eurexpress.org/ee/). In addition, this fragment overlaps with an orthologous region studied in the ENCODE project pilot phase (ENm005) [23], providing additional data on the locus. In order to screen this fragment systematically for enhancer activity, we generated a library (Figure 1b) of 142 overlapping clones (sizes: 1.5–4 kb) in a LacZ reporter lentiviral vector (LV) construct containing a minimal promoter (pRRLβLac, Figure 1a). Lentivectors are suitable for this kind of application due to their relatively large cloning capacity of around 10 kb between the LTRs without much of a drop in titer. The library was generated by partial digestion of the BAC with CvIJ and gaps were filled-in by cloning of PCR fragments. After exclusion of a 5.5 kb gap composed mostly of an LTR (chr16: 91,140,442-91,145,979 Mm8), the coverage of the library is >90%, with an average clone length of 2352 bp.

### In vivo screening by lentivector-mediated transgenesis

Lentiviral vectors can efficiently integrate into the genomes of early blastomeres following infection of zygotes from a wide variety of species including mice, rats, pigs, cows, and chickens [24], [25]. After injection of concentrated LV under the zona pellucida of a mouse fertilized oocyte, integration occurs at the 2- to 4-cell stage, resulting in usually one to a few but in some cases up to 15–20 proviral copies per transgenic animal [26]. From a routine injection session, fifty transgenic embryos with a given vector could be obtained. We thus decided to inject our library of lentiviral vectors in pools rather than individually, and to trace them back by PCR amplification of embryonic DNA, using primers specific for the library fragments contained in the proviruses. Vectors were produced separately by transient transfection of 293T cells and pooled during the concentration step. This appeared more suitable than the transfection of pooled plasmids, where competition between LV genomic RNAs for packaging during production, which could occur if some members of the library bore detrimental elements such as introns, cryptic polyA signals or RNA secondary structures, might introduce a bias. In parallel, each LV was titrated individually. As expected, vectors with larger inserts yielded lower titers, which in turn correlated with their less frequent representation in the transgenic embryos. LV titer is indeed a critical parameter, as in our hands vectors with a titer below 1×10^8^ TU/ml hardly yield any transgenic animals. After pooling, the infectious titers of individual vectors was in the range 1×10^8^ TU/ml, which predicted that each would be present in only a fraction of the embryos injected with the pool.

As a proof of principle, we first injected oocytes with a vector containing the well-characterized Sonic hedghehog limb enhancer [3], and performed LacZ staining on E11 embryos. All resulting transgenic embryos exhibited the expected limb bud domain staining (n = 30) (Figure 1c). Some embryos also showed some expression in other domains, but variable from one embryo to the other. This kind of variable random activity is also seen in embryos injected with an enhancer-less pRRLβLac, suggesting that it is due to some “enhancer trapping” effect (i.e. activation of the reporter by an endogenous enhancer located in the neighborhood of the transgene integration site, later referred to as “position effect”). It is more frequently seen when stocks with high MOI are used, and we therefore worked in conditions to minimize this. For example, under these conditions, the injection of the enhancer-less reporter yielded three LacZ positive embryos out of twenty transgenic ones (15%), two of which showed a broad and diffuse pattern, the third having staining in specific structures (Figure 1c). We attribute these cases to position effects, which do not impede on accurate identification of genuine signals coming from tissue-specific enhancers, since the ectopic signals associated with this "background" activity occurred stochastically, usually in restricted regions of the embryos and were not seen reproducibly in several embryos. We then proceeded with the LV library. In total, 8 pools of 10 or 20 vectors were injected (pools A to H, Table S2) representing a total of 109 clones (95 after exclusion of redundant clones, Table S3) and covering 173 kb, with no overlap. 81 (85% of injected) clones yielded integrants representing 162.7 kb (74% of the BAC). LacZ expression patterns were noted, and matched with the genotype of each embryo. Table 1 shows the results obtained with pool B as an example. The eight pools tested yielded 84 of 370 LacZ positive embryos with ∼2.3 transgenes per embryo (Table 2). Real time quantitative PCR data from pool A revealed that while most vectors were present in more than one embryos, each usually integrated as a single copy in a given embryo (data not shown). This effort allowed us to try and correlate LacZ expression patterns with the presence of particular LV clones. Candidate vectors thereby identified as potentially containing an enhancer were then re-injected individually to confirm activity.

**Table 1:** Genotyping Table.

	Clone										
Embryo number	5A5	5C7	5D6	5G1	5H8	5I7	6E6	6F7	7D7	5I2	Nb of different clones integrated
**59**	x	x			x			x	x		**5**
60		x						x			**2**
**61**	x	x	x	x	x			x	x		**7**
62		x	x								**2**
63					x						**1**
64				x							**1**
65											**0**
66		x			x			x			**3**
67											**0**
**68**	x	x		x	x			x	x		**6**
69									x		**1**
**70**		x							x		**2**
71	x			x							**2**
**72**	x	x						x	x		**4**
91	x	x							x		**3**
92					x			x	x		**3**
**93**	x	x	x	x	x			x	x		**7**
94				x	x			x	x		**4**
177	x	x	x						x		**4**
178		x	x								**2**
179					x				x		**2**
**180**	x	x	x	x	x			x	x		**7**
181					x			x	x		**3**
182											**0**
183					x				x		**2**
184	x								x		**2**
**185**	x				x				x		**3**
**186**	x	x			x			x	x		**5**
**187**	x	x	x	x	x			x	x		**7**
188											**0**
**189**	x										**1**
**190**	x	x	x		x			x	x		**6**
**191**	x		x					x	x		**4**
**192**	x	x									**2**
**193**	x	x	x	x	x				x		**6**
194					x				x		**2**
195											**0**
196	x							x	x		**3**
197		x		x				x	x		**4**
198		x	x		x			x	x		**5**
**199**					x			x			**2**
200	x				x			x	x		**4**
201			x					x	x		**3**
	**20**	**20**	**12**	**10**	**21**	**0**	**0**	**21**	**25**	**0**	

Example of a genotyping table. Pool B clones are displayed horizontally and embryo numbers vertically. X indicates that the embryo integrated the corresponding clone. Embryo numbers in bold face indicate LacZ positive animals. Candidate clones were identified by first looking for a repetition of a specific staining pattern among embryos injected with a pool of sequences. Within the subset of embryos that displayed the same pattern, we looked for common integrated clones. These fragments were the candidate enhancers to be injected individually for confirmation.

**Table 2:** Pools of lentivectors injected.

Pool	Number of vectors	LacZ positive embryos	Total embryos	Average number of different clones integrated	Average number of different clones integrated: LacZ embryos
pool A	9	5	31	3.1	5.0
pool B	10	16	43	3.1	4.0
pool C	10	2	39	0.5	3.0
pool D	10	21	40	4.8	7.0
pool E	10	4	42	0.4	2.0
pool F	20	16	52	2.0	3.8
pool G	20	12	54	0.9	2.3
pool H	20	8	88	2.2	6.1
					
Total	109	84	389	2.1	4.2

Summary of injected pools.

### Identification of non-conserved and conserved enhancers

**Figure 2:**
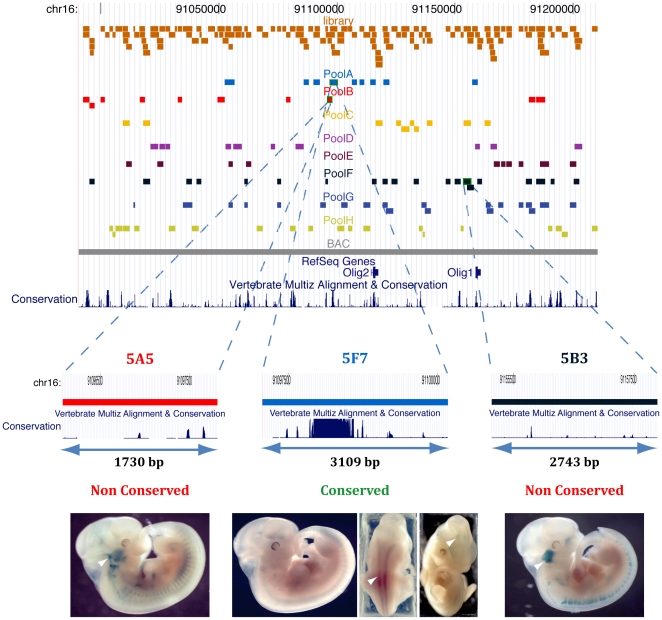
Conserved and Non-conserved enhancers. Genomic location of the 3 identified enhancers in the UCSC genome browser. Middle: Close-up on the 3 enhancers, showing that 2 are not significantly evolutionarily conserved. Bottom: expression pattern of characteristic embryos transgenic for the corresponding clone. Arrowheads highlight expression in the neural tube and trigeminal ganglion.

In pool B, 3 embryos (185,189,190) were found to have staining in the trigeminal ganglion, all of which had integrated LV clone 5A5 (Mm8 Chr16: 91096078-91097808), pointing to the corresponding DNA insert as a good enhancer candidate. When this clone was re-injected individually, 4/6 (66%) embryos exhibited staining in the trigeminal ganglion (Figure 2). This proportion was significantly (p = 4.9×10^−4^) above the background staining of this anatomical region in the totality of the transgenic embryos (6.6%). Noteworthy, the BAC fragment contained in this clone is not evolutionarily conserved based on current annotations and detection methods (UCSC genome browser [27], Vertebrate Multiz Alignment & PhastCons Conservation [28],). Another clone (5B3, from pool F, Mm8 Chr16: 91155139–91157882), containing an insert located 57 kb downstream of 5A5, similarly induced expression in the trigeminal ganglion (Figure 2), although in this case the penetrance of the phenotype was lower (41%, n = 22), albeit highly significant (p = 8×10^−4^). This enhancer was also not evolutionarily conserved (Figure 2).

**Figure 3:**
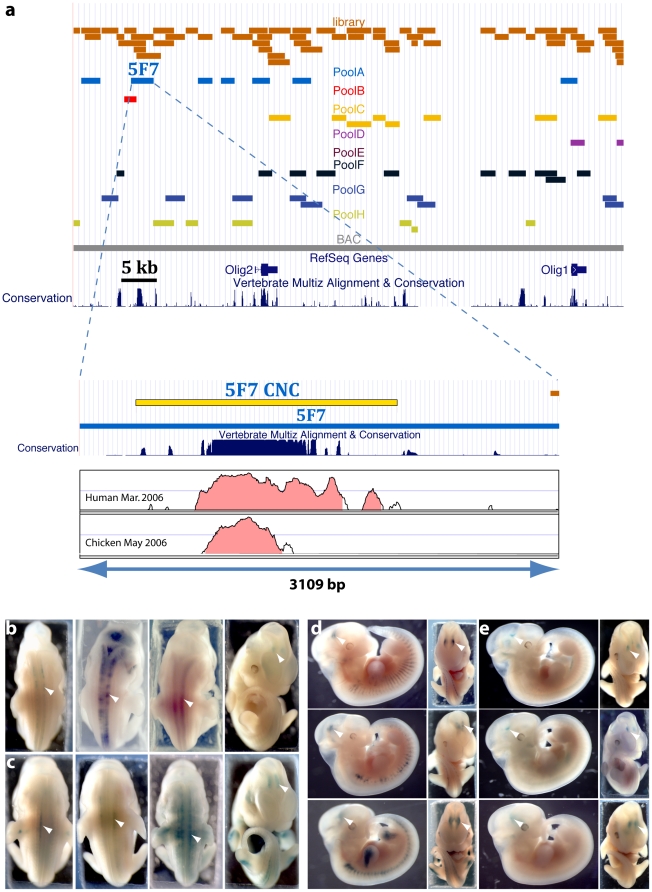
Characterization of enhancer 5F7. (A) Genomic location of clone 5F7 (pool A). Bottom: Close view of genomic location of 5F7 with the UCSC vertebrate conservation track and the VISTA alignment of human and chicken orthologous regions. The yellow box shows the boundaries of the conserved fragment that we subsequently cloned (5F7 CNC). (B) Embryos injected with clone 5F7 individually express the reporter in the neural tube and brain (arrowheads). 3^rd^ and 4^th^ images are back and front views of the same embryo. (C) Embryos injected with clone 5F7 CNC individually also express the reporter in the neural tube and brain. 3^rd^ and 4^th^ images are back and front views of the same embryo. Arrowheads highlight expression in the neural tube and trigeminal ganglion. (D) Lateral and frontal views of 3 embryos injected with chicken 5F7 orthologous sequence displaying posterior diencephalon staining (arrowheads). (E) Lateral and frontal views of 3 embryos injected with human 5F7 orthologous sequence displaying posterior diencephalon staining (arrowheads).

In contrast, the third transcriptional enhancer identified carried by clone 5F7 (Mm8 Chr16: 91097068–91100177), from a genomic fragment located 17.3 kb upstream of *Olig*2, is highly conserved (Figure 3a). The corresponding vector induced consistent staining in the neural tube (71% embryos, n = 7, p = 8*10^−5^) and brain (57%, n = 7) (Figure 3b). While 5F7 contains a 1.5 kb block of strong evolutionary conservation (Figure 3a), this region was found to yield only small peaks of DNase I hypersensitivity in human cell lines (ENCODE data; [23]). The specificity of this enhancer is particularly interesting since it could participate in controlling the expression pattern of the *Olig* genes. However, when transduced in cell lines, 5F7 induced LacZ expression in all cells tested whether they expressed *Olig* genes (U87, HL60) or not (K562, 293T, HCT116) (not shown).

When oocytes were injected with a minimal promoter LacZ vector containing only the conserved segment of clone 5F7 (5F7 CNC, Figure 3a), the resulting embryos again showed neural tube staining (54% embryos, n = 24, p = 1*10^−4^, Figure 3c). This 1.5 kb genomic is thus sufficient to confer the phenotype. Interestingly, it was predicted to be functional in a recent *in silico* study based on sequence conservation and density of potential transcription factor binding sites [29].

### Orthologous 5F7 sequences demonstrate CNS enhancer activity

We further characterised this enhancer by testing the activity of orthologous human (hg18: chr21:33′301′930-33′304′097, 2168 bp) and chicken sequences (710 bp, galGal3: chr1:108,639,445–108,640,154). Orthologous coordinates of human and chicken sequences were obtained through the UCSC genome browser and the segments were PCR amplified using primers within the intervals (Vista alignment is shown in Figure 3a). We find that both human and chicken 5F7 elements display strong enhancer activity in the CNS. The most penetrant phenotype was staining in the posterior diencephalon with 94% (p<10^−10^, n = 17) and 81% (p<10^−10^, n = 16) of embryos showing this pattern with the chicken (Figure 3d) and human (Figure 3e) elements, respectively. Expression of the reporter was also present in the neural tube as with the mouse element, although with a lower frequency (52% for the chicken element, n = 17, p = 6.9*10^−6^; 31% for the human, n = 16, p = 7.7*10^−3^). Collectively, these data identify clone 5F7 as a strong CNS enhancer with reproducible staining in the posterior diencephalon and neural tube. Both phenotypes were seen for human, mouse and chicken orthologs, but the penetrance varied depending on the species of origin (Table 3). Human and chicken elements were almost identical, while the mouse ortholog drove expression of the reporter more frequently in the neural tube and less frequently in the posterior diencephalon. This could reflect a different interpretation of a functional element due to species-specific transcription factors. Alternatively, it is possible that parts of the functional module are missing for some of our constructs, since we do not know the exact boundaries of the activity. For example, the mouse element could span a larger genomic fragment than its human and chicken counterparts; and our construct could thus lack part of the required sequences.

**Table 3:** Frequencies of phenotypes observed for clone 5F7.

	Mouse 5F7 (n = 7)	Mouse 5F7 CNC (n = 24)	Human 5F7 (n = 16)	Chicken 5F7 (n = 17)
Neural tube	57%	54%	31%	52%
Posterior Diencephalon	14%	13%	81%	94%

Frequencies of neural tube and posterior diencephalon staining for embryos injected with: Mm5F7, Mm5F7 CNC, Gal5F7, Hs5F7.

### *Olig* gene expression overlaps with 5F7-LacZ stainings

**Figure 4:**
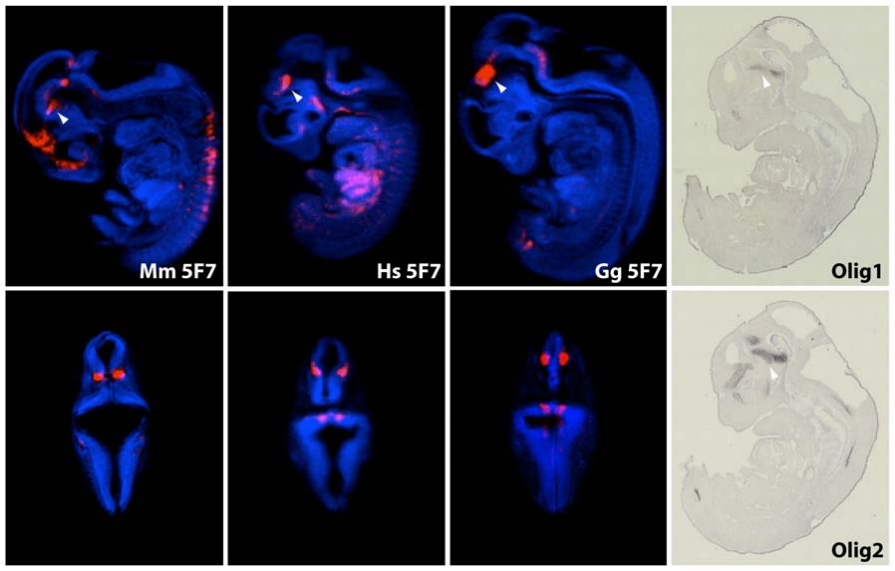
Optical projection tomography of 5F7-LacZ stained embryos. Optical projection tomography (OPT) images of selected LacZ stained embryos. One representative embryo for each orthologous mouse human and chicken enhancer was selected. Arrowheads highlight expression in the trigeminal ganglion. Top: sagittal sections. Bottom: frontal sections. (Right) In situ hybridisations for *Olig*1 and *Olig*2 genes.

**Figure 5:**
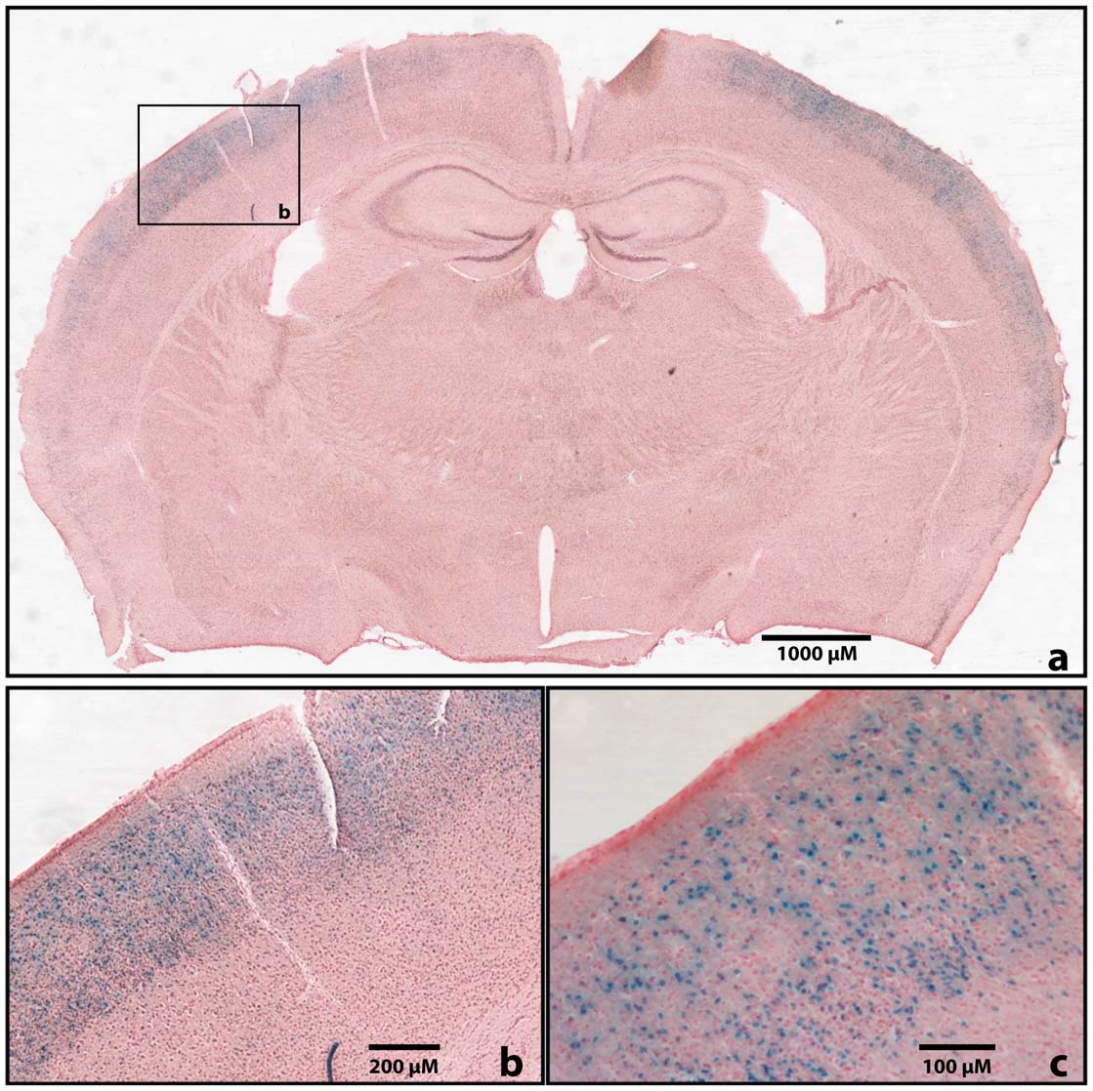
Expression of 5F7-LacZ in adult brain. (A-B-C) Brain sections of adult mice transgenic for 5F7-LacZ at different magnifications. Expression of the reporter is specific to layers I, II, and III of the cortex. Cell nuclei are visible at higher magnification (C).

Since the activity of clone 5F7 is specific to the CNS, in the neural tube and posterior diencephalon, we hypothesized that it could be responsible for part of the expression pattern of the nearby *Olig* genes. *Olig1* and *Olig2* (oligodendrocyte transcription factor) are bHLH transcription factors [22] that promote formation and maturation of oligodendrocytes. They cooperate to establish the progenitors of motor neurons (pMNs) in the embryonic neural tube. Since *Olig*1 and *Olig*2 are clustered and act in concert to differentiate oligodendrocytes, it is possible they belong to the same regulatory landscape [30], [31] and are co-regulated by shared *cis*-elements. To test whether the expression of *Olig* genes overlapped with our LacZ stainings of clone 5F7, we performed in situ hybridization at E11.5 on histological sections (Figure 4). We then compared these ISH patterns with virtual sections of 5F7 LacZ stainings generated by optical projection tomography (OPT) (Figure 4). Remarkably, we found that 5F7-LacZ and *Olig* genes shared a very specific expression domain in the posterior diencephalon, suggesting that the identified transcriptional enhancer could be directing expression of *Olig* genes in this tissue. While the human and chicken elements drive expression of the reporter in very similar domains, the domain elicited by the mouse sequence seems more extensive. The activities of the orthologous elements may be slightly different or interpreted in a different way by the murine transcriptional machinery. Moreover, we cannot be certain the enhancers behave exactly as they would in their normal genomic context. We further probed the activity of enhancer 5F7 by generating adult mice transgenic for 5F7-LacZ. Expression of the reporter was investigated on adult brain sections and was observed in neurons of layers I, II, and III of the cortex (Figure 5). Thus, 5F7 acts as an enhancer in neural tissue both during embryonic development and adulthood.

### Clone 5F7 contacts the *OLIG2* promoter

**Figure 6:**
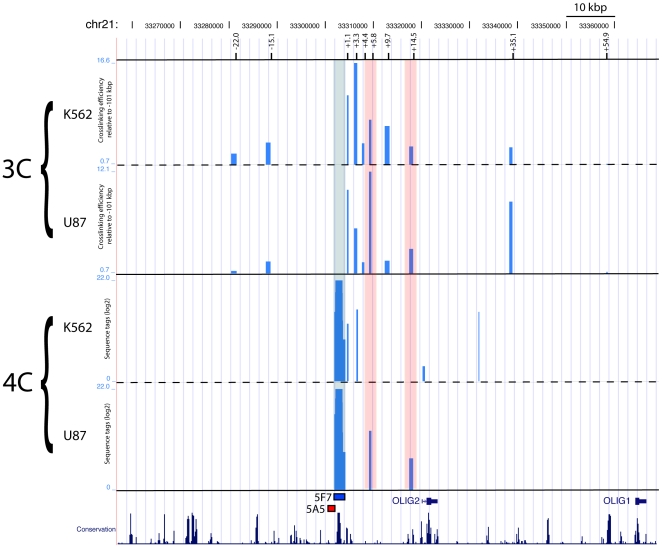
3C and 4C interactions between 5F7 and Olig2. 3C and 4C interactions between 5F7 and *OLIG2* in K562 and U87 cells. 3C: Crosslinking efficiency was measured for each probes relative to a fully digested/ligated BAC (RP11-760B14; chr21: 33199567–33414452) and then ploted relative to a probe located 102 kb upstream of 5F7 where crosslinking is expected to be at background levels (the −102 kbp site is not shown here). 4C: A DpnII fragment containing clone 5F7 was used as bait. The vertical lines correspond to the log2 of the number of tags recovered by the bait in K562 and U87 cells. The peak at the bait represents self-ligation of 5F7.The position of 5F7 is indicated by a light blue strip whereas the U87 4C-specific interactions are indicated by light-red strips.

If clone 5F7 indeed regulates the *OLIG* genes, we hypothesized that they may interact in chromosomes. We tested this hypothesis using circular chromosome conformation capture (4C) in U87 (human glioblastoma) and K562 (human erythromyeloblastoid leukemia) cells with clone 5F7 as bait (5F7-DpnII). The library was generated by digestion with DpnII. We found that the bait interacted with a fragment just upstream of the *OLIG2* transcription start site (Figure 6), consistent with the hypothesis that the enhancer contributes to the expression pattern of *OLIG2*. In K562 cells, that do not express *OLIG2*, only 9 tags were recovered from the single intron of *OLIG2*, 250 bp downstream of the described *OLIG2* basal promoter [32], [33]. In contrast, *OLIG2*-expressing U87 cells [34], showed a much stronger interaction between 5F7 and *OLIG2*, with 13-fold more tags (118 tags) recovered (after normalization for the total number of tags). The fragment recovered from the U87 cells (hg18: chr21: 33317543–33318140) is slightly more centromeric (2.1 kb and 7 DpnII sites separate the U87 fragment from the K562 fragment) than the K562 fragment (hg18: chr21: 33320303–33320554), and overlaps with the promoter of *OLIG2*. The two cell lines thus show different chromatin dynamics that could be reflecting that fact that one expresses *OLIG2* (U87) while the other doesn't (K562). The relative proximity between *OLIG2* and 5F7 (14.5 kb) raises the possibility that the observed interactions stem from a proximity effect rather than from an active process. In order to further discriminate between these possibilities we performed quantitative 3C using TaqMan assays in biological duplicates (Figure 6). We designed-dual labeled probes encompassing the potential ligation products between 5F7 and a series of eleven DpnII fragments surrounding the CNC (Table S4). Crosslinking efficiency decays relatively fast from 10 fold (+1.1 kbp) to 1.8 fold (+4.4 kbp) in U87 suggesting that the proximity effect of crosslinking does not extend far from the bait as observed earlier under the same conditions [35]. Interestingly, crosslinking efficiency increases again at +5.8 kbp from 5F7 (12 fold) recapitulating the interaction observed in 4C. A smaller peak of enrichment (3.5 fold) is present at +14.5 kbp corresponding to the promoter region of *OLIG2*, which was shown to interact with 5F7 in 4C. Another site (+35.1 kb), between *OLIG2* and *OLIG1*, shows strong association (8.7 fold) with 5F7 in U87 cells. A significantly weaker interaction, but still above background, is observed at this site in K562 (3.4 fold). The potential function of this interaction is unclear although it may suggest that 5F7 is capable to fold over *OLIG2*. Interestingly this site lies near a block of evolutionary conservation. A similar interaction however was not detected in 4C. In addition, we could not design appropriate TaqMan probes for the 4C DpnII fragment identified in K562 right upstream of *OLIG2* (+16.5 kbp). Overall, these data suggest that the interaction between 5F7 and *OLIG2* in U87 cells is genuine and not an artifact due to proximity. This observation adds further support to the conclusion that the identified enhancer could be regulating *OLIG2*.

## Discussion

We present a rapid and unbiased *in vivo* method to screen a large genomic fragment for enhancer activity. The high efficiency of lentiviral vector-mediated transgenesis [26] enables testing of many sequences in a single experiment. Moreover, the method bypasses time-consuming mouse breeding since it does not need the generation and maintenance of transgenic lines, but is instead based on the analysis of F0 embryos. The method described here substantially diminishes the number of oocyte injections and foster mice and thus increases the throughput compared to single construct injections [19]. Our demonstration that injecting pools of up to 20 different lentiviral vectors leads to the successful identification of transcriptional enhancers allows the scale-up of this enhancer screen covering up to megabases of DNA.

We have extensively screened a mouse BAC for enhancer activity, with over 74% of the total sequence tested. To our knowledge, this is the first broad unbiased (i.e. not driven by evolutionary conservation) screen for transcriptional enhancers in transgenic mice. We identify 3 enhancers with a high degree of confidence, the most robust of which drives expression of the reporter in the posterior diencephalon and neural tube. Importantly, of the three identified enhancers, only one is strongly evolutionarily conserved. The two other regulatory elements show no detectable sequence conservation whatsoever and would not have been uncovered in a conservation-based candidate approach. This observation indicates that exhaustive screens for functional elements should not be restricted to conserved DNA elements. Moreover, while current annotation of the mouse genome (NCBI37-mm9) does not display predicted transcription factor binding sites, the human orthologous fragment of enhancer 5F7 harbours an abundance of predicted binding sites (FoxC1, Oct-B1, POU3F2). It is possible that even the non-conserved elements may contain a short sequence of conservation that is responsible for enhancing activities, particularly since the typical transcription factor binding site is just a few nucleotides-long. Interestingly, the two non-conserved enhancers, separated by only 57 kb, displayed the same pattern of reporter expression in the trigeminal ganglion. They could represent « shadow enhancers » with overlapping activities [36], but it remains unknown whether the target gene of these enhancers is *Olig* or a more distant or unannotated gene.

Since we screened a BAC mapped within an orthologous fragment studied in the ENCODE project pilot phase, we asked whether our identified conserved enhancer 5F7 carried annotations suggestive of function. Human 5F7 does not show any significant DNaseI hypesensitivity in the seven cell lines tested (CD4+ T cells, GM06990 lymphoblastoid, HeLa S3 cervical carcinoma, HepG2 liver carcinoma, H9 human embryonic stem, IMR90 human fibroblast, K562 myeloid leukemia-derived). Interestingly, human 5F7 is mostly covered by repressive chromatin marks (H3K27me3 mainly) in all cell lines investigated by ENCODE (erythroleukaemia, umbilical vein endothelial, skeletal muscle myoblast, mammary epithelial, lymphoblastoid, embryonic stem, epidermal keratinocyte, lung fibroblast). However, the most conserved part of human 5F7 is marked by monomethylation on lysine 4 of histone H3 (H3K4me1) in embryonic stem cells (H1-hESC), a modification associated with enhancers [37], [38]. This suggests that the locus is tightly regulated and mostly repressed but can be activated in a specific spatio-temporal manner. Such a tight control pattern would be compatible with the likely regulation of *OLIG* genes. These data should be treated with caution however as they originate from non-neural human cell lines that likely differ in their regulation of this locus compared to LacZ positive cells in our E11 murine embryos. We also looked at p300 binding sites in forebrain, midbrain and limbs of E11 mouse embryos (data from [39]), but none of our identified enhancers overlapped with a peak of p300 binding in these tissues.

The ENCODE project pilot phase had previously described several functional regions that showed no evidence of evolutionary constraint [23]. Likewise, another report had subsequently suggested that non-conserved elements could also harbour enhancer activities in zebrafish transgenics [20], but a broad unbiased screen had not so far been conducted in mice. Here, we provide further evidence that non-conserved sequences with enhancer activity exist. This observation has important implications regarding the annotation of genomes and the identification of disease-related variation. It is noteworthy that our study presented two limitations precluding the exhaustive identification of enhancers in the DNA region under study. First, we concentrated our analysis on a narrow window of embryonic development. Second, overlapping signals may have masked the activity of some discrete enhancers.

To increase the likelihood of discovering sequences potentially associated with human disorders, we set out to study a region syntenic with human chromosome 21 that harbours the *OLIG1* and *OLIG2* genes. These genes are specifically expressed in the CNS, hence their dysregulation is potentially involved in Down Syndrome. A recent study in a mouse model of Down Syndrome confirmed that *Olig* genes triplication indeed causes neurological phenotypes [40]. Moreover, *OLIG2* deregulation has been associated with disorders such as schizophrenia [41], [42] and Alzheimer's disease [43]. Our in situ hybridisation and chromosome conformation capture data support the hypothesis that enhancer 5F7 contributes to the expression pattern of *OLIG* genes in the posterior diencephalon but could also be regulating other more distant genes. The specificity of this CNS transcriptional enhancer slightly differed between the human, chicken and mouse orthologous sequences. All three highlighted the posterior diencephalon and the neural tube. However, the human and chicken elements displayed very similar staining with a higher frequency of diencephalon staining and lower frequency of neural tube staining, relative to the mouse element. These differences could be explained by an inaccurate “reading” of foreign DNA fragments by the murine transcriptional machinery. However, the recent study of a transchromosomal mouse carrying human chromosome 21 showed that the foreign chromosome could be recognized and interpreted in the appropriate spatio-temporal manner by the host machinery. In hepatocytes of this mouse, the human chromosome was recognised by murine transcription factors to dictate accurate gene-expression despite the lack of conservation of certain DNA binding motifs; showing that adequate instructions to direct species-specific transcription must be embedded in the genetic sequence [44]. Alternatively, the differences we observe could be evolutionarily relevant and represent species-specific differential regulation. A recent example of such differences was reported for an enhancer gaining a limb expression domain in human relative to chimpanzee [45]. However, the high relatedness of the expression patterns induced by all three orthologous 5F7 elements strongly suggests a conserved role for this enhancer in the three species. Furthermore, the combination of all the patterns seen with the three enhancers includes all the patterns seen in *Olig1* and *Olig2* in situ hybridizations. For example, the less penetrant LacZ neural tube and hindbrain domains are visible in the *Olig2* in situ hybridization. How different activities of this enhancer are generated with respect to *Olig1* or *Olig2* at the original locus in different tissues is not known and could be dependant on other regulatory influences coming from additional *cis*-acting elements.

It is becoming increasingly apparent that sequence conservation alone is not a sufficient criterion to predict all regulatory elements and that other features can facilitate the identification of functional sequences. For example, a recent study showed that p300 association accurately predicted tissue-specific activity of enhancers [39], while evolutionary conservation of the three-dimensional structure of DNA has also been proposed as a marker of functional elements [46]. It is likely that a combination of chromatin marks [37], [47], bound proteins [39], DNaseI hypersentivity [48], three-dimensional structure [46] and sequence conservation criteria along with other yet unknown parameters will be required to improve the prediction of regulatory elements. The method we present here could be scaled up to cover large chromosomal regions, and determine what fraction of the conserved and non-conserved genome has regulatory potential.

## Materials and Methods

### Library construction

Murine BAC RP23-356P18 (219.6 kb) was obtained from CHORI (http://www.chori.org/) and partially digested with CviJ. Fragments of 2–4 kb were purified and cloned in pRRLβLac (Trono lab: http://tronolab.epfl.ch/). Clones were end sequenced and re-mapped to the BAC. Gaps were filled-in by cloning PCR fragments. The library contains 142 clones and covers more than 90% of the BAC (Table S1), after exclusion of the large gap containing an LTR repeat.

### Lentivector-mediated transgenesis

Lentiviral vectors were generated by co-transfecting the transfer vector with PMD2G and R8.74 plasmids (Trono lab: http://tronolab.epfl.ch/) in 293T cells. One 50–60% confluent 15 cm diameter petri dish was used for each fragment. 3 collections of 14 ml of supernatant were performed, at 24 h (transfection medium changed after 16 h), 36 and 48 h after transfection. 7 ml (pools of 10) or 3.5 ml (pools of 20) of each vector were pooled (70 ml) for ultracentrifugation in two 35 ml-tubes, the rest of the supernatant was used to prepare individual vectors. Pools were resuspended in 3×20 µl for injection, individual vectors in 3×10 µl. For each clone of the library F0 transgenic embryos were generated by perivitelline injection of lentivectors in mouse fertilised oocytes as described in [26]. Briefly, 50–150 oocytes were injected and transferred to foster mothers (16 embryos/foster). Fosters were sacrificed to recover 5–14 E11.5 embryos/foster. 31 to 69 embryos were recovered for pools, ∼30 or more for individual vectors. Embryos were stained for LacZ in 0.8 ml assay mix in 24-well plates. Staining pattern were identified and photographed for analysis.

### Genotyping

A PCR assay was developed to genotype each injected clone of the library individually using one clone specific primer (Table S2) and the BGdown primer (AGCAATAGATGGCTCTGCCCTGAC) in the beta globin minimal promoter, which is common to all. Each embryo was genotyped for all clones of the pool potentially integrated.

### Statistical analysis

P values for staining patterns are obtained by Fisher's exact test on a 2×2 table. For a particular construct, frequency of observed pattern is compared with frequency of observing this pattern in all other beta galacotosidase embryos having received other random fragments of the library.

### Optical projection tomography

To further characterize LacZ stainings, Optical Projection Tomography (OPT) (http://www.bioptonics.com/) was used to generate 3D reconstruction and virtual sections of embryos.

### In situ hybridization

RNA Probe Synthesis: Digoxygenin-tagged RNA probes were generated from the DNA templates T3961 and MH_QR465, for *Olig*1 and *Olig*2 respectively. T3961 was obtained through the Eurexpress Consortium (www.eurexpress.org) and MH_QR465 was a kind gift of Pr. G. Eichele (MPI, Göttingen). In vitro transcription was carried out in 1 mM rATP, rCTP, rGTP, 0.65 mM rUTP, and 0.35 mM digoxigenin-UTP (Roche labeling kit), 1 µl of ribonuclease inhibitor (40 U/µl MBI Promega), 0.5 µg of DNA template, 0.5 µl of RNA polymerase (T7: 50 U/l, SP6: 20 U/l, both New England Biolabs), in 20 µl.

In situ hybridization: E11.5 mouse embryos were embedded in OCT. Embryos were sagitally sectioned at 25 um thickness on superfrost slides. Tissue sections were then fixed in 4% paraformaldehyde, acetylated and ISH were performed as described previously [49].

### Circular chromosome conformation capture (4C)

Chromosome conformation capture was performed as described [50] with the following modifications. Cells were grown in 50 ml RPMI medium (Invitrogen) supplemented with 10% heat inactivated fetal bovine serum and 100 µg streptomycin/penicillin at a concentration of about 2×10^5^ cells/ml (1×10^7^ cells). Crosslinking with formaldehyde (1% v/v) was allowed to proceed for 10 minutes at room temperature directly in the cell media prior to quenching with 125 mM glycine. Cells were washed twice in ice-cold PBS and lysed for 1 hour on ice with mild stirring in 20 ml 1xTBS-Tween (10 mM Tris-HCl pH 7.5, 3 mM CaCl2, 2 mM MgCl2, 15 mM NaCl, 0.5% v/v Tween 40) supplemented with protease inhibitor (Complete, Roche) and 5 mM PMSF. The cell lysate was homogenized with 15 strokes in a Douncer (‘A’ or tight pestle) and washed with PBS (centrifugation: 1′200 g at 4°C for 10 minutes). The lysate was subsequently resuspended in 5 ml 25% (w/v) sucrose-TBS and underlayed with 5 ml 50% (w/v) sucrose-TBS. Nuclei were pelleted for 20 min (4600 g at 4°C), washed under the same conditions with 5 ml 25% (w/v) sucrose-TBS and resuspended in 500 µl 1.2 x DpnII restriction buffer. Restriction with DpnII, ligation, crosslink reversal and DNA purification were carried out as described earlier [35].

The 4C library was generated from 200 ng of ligated DNA with two successive rounds of PCR amplification using 2 nested pairs of primers. The PCR (20pmoles of round A primers) were performed under the following conditions during RoundA: 98°C for 30 s, 34 cycles of 98°C for 10s/65°C for 30s/72°C for 90 s and followed by a final elongation step at 72°C for 3 min. A 1/100 dilution of round A DNA is then amplified with 40pmoles of round B primers (94°C for 3 min, 32 cycles of 94°C for 30ss/65°C for 30s/72°C for 90 s and followed by a final elongation step at 72°C for 3 min). The primers used during the second round of amplification have additional nucleotides at their 5′ end (5′-AATGATACGGCGACCACCGA and 5′-CAAGCAGAAGACGGCATACGA). These are required for DNA colony amplification on the cluster station as part of the Illumina Genome Analyzer high-throughput sequencing procedure. The library was gel purified to reduce the amount of DNA originating for self-ligation of the DpnII restricted bait. Sequencing was carried out at Fasteris life sciences (http://www.fasteris.com) using an Illunima Genome Analyzer. The sequencing primers were designed to anneal just upstream of the DpnII (GATC) restriction site on one side of the bait. Hence all sequences start with GATC.

### Chromosome conformation capture (3C)

For quantitative Taqman PCR, we designed 11 assays comprising the PCR primers and a dual-labeled probe sitting at the predicted DpnII junction between the target and bait regions (Table S4). PCR reactions were set up as described earlier [35]. Technical triplicates were performed on biological duplicates both in K562 and in U87. For the 3C samples, 200 ng of DNA was used per well, and for the BAC RP11-760B14 (chr21: 33199567–33414452), 5 ng of digested and randomly ligated DNA was used. Normalization for each assay was performed using the values obtained from BAC experiment. Enrichment was calculated with respect to the most centromeric probes (−102 kb), which showed very low levels of interaction.

## Supporting Information

Table S1 Coordinates of the 142 clones of the library. All map to Mmu16 and positions are given for mm8 (Feb. 2006) mouse genome assembly.
(PDF)

Table S2 All clones injected, and the sequence of the primer used to genotype. The orientation of each clone is indicated. “+” denotes that the sequence upstream of the lacZ reporter is the positive strand. “−” indicates the negative strand (Mm8, UCSC genome browser http://genome.ucsc.edu/).
(PDF)

Table S3 Summary of all injected clones. Coordinates, ID, Pool, integration status.
(PDF)

Table S4 3C primers and probe.
(PDF)
