# Supplementary material for: A Systematic Enhancer Screen Using Lentivector Transgenesis Identifies Conserved and Non-Conserved Functional Elements at the Olig1 and Olig2 Locus
Source: PLoS One. 2010 Dec 29;5(12):e15741. doi: 10.1371/journal.pone.0015741 (PMC3012086; doi:10.1371/journal.pone.0015741)
Supplement: Table S1 — Coordinates of the 142 clones of the library. All map to Mmu16 and positions are given for mm8 (Feb. 2006) mouse genome assembly. (PDF) [file pone.0015741.s001.pdf]

Table S1

Chr 16 (Mm8) start	Chr 16 (Mm8) end	Clone ID
90992462	90995219	5H8
90992487	90995219	5B7
90995220	90997337	5I3
90995220	90997339	5I2
90995502	90997083	5I4
90999956	91001783	5G1
91001974	91003811	4bis
91003792	91005208	6c
91005029	91006312	5c
91006178	91009107	7'
91009313	91011553	5F5
91009408	91012175	5L1
91009413	91011740	5D4
91010795	91013098	5B6
91013753	91014547	9E11
91014243	91016402	9'
91016413	91018677	9A9
91016502	91018759	6F7
91017256	91019321	8G10
91017747	91019919	5K5
91017932	91020851	5B2
91021023	91024244	5L3
91021143	91024756	5L5
91023842	91026455	8E3
91023959	91026413	5K4
91024807	91027134	7C5
91027385	91029416	6E5
91028867	91031500	11c
91031401	91032315	11d
91032548	91034365	5C7
91034777	91038675	9F12
91036252	91038654	9B3
91038522	91041473	14'
91040534	91042635	9B6
91041798	91042701	9H12
91042573	91045525	15'
91045517	91048321	9D7
91048128	91049593	17'
91049457	91052518	5D6
91050089	91051982	5L8
91051619	91054051	8F7
91052568	91056665	6L7
91052759	91055215	5J6
91054035	91055972	6E8
91056171	91059711	7E9
91056984	91059408	6K7
91059596	91060795	18c
91060774	91062326	8G2
91061492	91063760	8A2
91065968	91068486	20d
91068464	91070853	21
91071964	91073765	8B5
91074712	91076395	8G1
91076912	91078546	24
91078438	91080339	7D7
91080093	91082418	25
91082413	91085986	5H4
91082802	91085986	8H5
91084287	91087566	8F9
91085935	91088116	6L4
91087971	91089883	26
91090067	91092760	5I6
91090560	91092790	8H3
91092940	91095282	9E7
91095033	91096114	28
91095283	91099109	9A4
91096078	91097808	5A5
91097068	91100177	5F7
91097434	91099130	Mm5F7CNC
91097822	91101241	10B3

91100121	91103069	29
91101872	91104576	9H6
91104717	91106432	30c
91106384	91108466	6D7
91109637	91111538	6K1
91111221	91114051	31bis
91113549	91116166	10A4
91113986	91116489	6B2
91114735	91116792	5B1
91114879	91116792	5G5
91116184	91118570	10A7
91116258	91119250	7G5
91116345	91119382	5A3
91119296	91121563	7C10
91119649	91122250	5D2
91119826	91122275	8C10
91120775	91123842	10A3
91123023	91125665	9B7
91125129	91127152	5D7
91127226	91130678	6G5
91127458	91129681	9D11
91130945	91132689	5A1
91130945	91132689	5D1
91132446	91134607	5K8
91132624	91134636	5F2
91134640	91136328	33
91135691	91137979	10B7
91136300	91137194	34
91137124	91139662	10A2
91138022	91140442	6J6
91145978	91148056	9C8
91147945	91149943	36'
91149913	91152319	37
91152306	91153625	38
91153618	91156723	5L4
91155139	91157882	5B3
91155228	91157840	5C3
91157209	91159549	5A7
91158569	91160533	6L1
91159441	91161886	10B2
91159456	91161383	8E11
91162463	91165047	6F4
91163066	91165057	7G11
91163375	91166325	6E1
91164390	91166475	10A1
91164838	91167002	7F7
91165080	91167632	6B1
91166522	91168984	5G6
91166583	91168972	5G7
91168261	91170385	9G6
91169486	91171384	5C5
91171699	91174038	8B10
91171825	91174065	8A1
91176911	91178881	8D5
91179971	91182599	45
91181242	91184052	5I7
91184260	91188053	6E6
91184946	91187860	7B8
91184993	91187204	8D6
91185129	91187717	8D10
91186024	91189770	8D4
91189427	91191182	55
91190549	91192157	8H4
91192647	91195233	53
91195094	91197673	42d
91198559	91201334	7E1
91200259	91202576	5F6
91200560	91203773	5L2
91200888	91203018	9E12
91208048	91210312	54
