# Supplementary material for: A Systematic Enhancer Screen Using Lentivector Transgenesis Identifies Conserved and Non-Conserved Functional Elements at the Olig1 and Olig2 Locus
Source: PLoS One. 2010 Dec 29;5(12):e15741. doi: 10.1371/journal.pone.0015741 (PMC3012086; doi:10.1371/journal.pone.0015741)
Supplement: Table S2 — All clones injected, and the sequence of the primer used to genotype. The orientation of each clone is indicated. “+” denotes that the sequence upstream of the lacZ reporter is the positive strand. “−” indicates the negative strand (Mm8, UCSC genome browser http://genome.ucsc.edu/). (PDF) [file pone.0015741.s002.pdf]

Table S2

Clone ID	Genotyping primer	Orientation	Pool
6L7	AGAGGGGAGAGGAGGGCAGA	-	A
6L4	CGATGACAGGCCTGCTAATGAC	-	A
5I6	CATGCTTCTGTCAAGAGGCACTCC	+	A
5F7	TCCAATGTACAGCCCTGGCCTA	-	A
6D7	TTTCTGTCGTGTGTCTATGCCATTACT	-	A
6K1	GCATTTCAGTGGCCAGGATCA	+	A
6B2	GCGGGAACAATGTGCTTTTCCT	-	A
5D2	TCCTTGGACCCGAGTACCTGAA	+	A
5A7	CCCTAAAGCTCCCTCCCTTGC	+	A
5H8	GGTCCCTCTGTTTCCCACCTCT	-	B
5I2	CCTAATCACGCGGAAATCTTGC	+	B
5G1	TATCGTGGGTGTGCCAGACACT	+	B
6F7	ATTCCCCTGGGTAGGCAGAGAG	-	B
5C7	TGAGCAGGTAGAAAACCCTTTCATTC	-	B
5D6	AAAGGGTCTATCCTGAGCCTTGC	-	B
7D7	CCCGTCAGTGTTCAGGGAGAAG	-	B
5A5	CACGGCCCTAGGACTGTTTTGT	-	B
5I7	TCATTGACCTCTTTAGGCTTTCTTCA	-	B
6E6	GGGGTTTGAAGACTGCTGGCTA	+	B
5L1	ATCCAGGTTAATGCGGGGACTT	-	C
5B2	TCTACGGCAGAGTCGTGGTGAG	+	C
5A3	GGCTAGCTCTGGGACAAGAACG	+	C
5D7	GCTTTCTGGCAGCTACAAAAGGA	+	C
6G5	TGCTGCAATGAAAGTCAGACAAA	+	C
5A1	GCCATATGCCAGGTGGAGAAAG	-	C
5F2	TCTTCCTTTGTGTCCGCATTGA	+	C
6J6	GGGCCCTCTCCTAAAAGCATGT	-	C
5L4	AAAGCTCCACCTGAGGGTGACA	+	C
6F4	GAAGGACTTTCCTCCAGCCACA	+	C
5l3	AGGGTAGGGTAAGGCATGCACA	-	D
7C5	AAAGCAGAGTCCGGGATTGTTG	-	D
6E5	GACTCCGCTTTGAAGCAGTTCC	+	D
5J6	CGAGTAATGGAAGCCCAGGTTG	+	D
7E9	GGTCCAGCCCTGTGTTTAGCA	+	D
8B5	CCATCACGTAAGTTCCCGCATT	+	D
5H4	CATCACTCCCTTGCCTCTCTCC	-	D
6L1	CTGCTGCTGCTGTTCCTCTTTG	-	D
6B1	GGGGATGCTGGTGTAAAGGAGA	-	D
5L2	CCCCAACAACTAGCAAATGAGTCC	+	D
5B6	GGGGCTACTCTATCCACCAGCAT	+	E
5K4	ATCGTGCTTTGAGCCTCTCTCCT	+	E
6E8	AGGGGTCAGGTATCTGCTGCTTT	-	E
8A2	CTGGCAGGGATGGAGACAAAAG	+	E
5G6	CCCATTGCTTACCTGTGACTTGC	-	E
5C5	TTCCATGACCACATTCCTGCTTT	-	E
8B10	GATGCAGAGTTGCAGGTTGAACA	+	E
8D5	TTCTAGCATGGCCTCAGGATGTG	-	E
8D4	GAGGTTGAAGGGTGCCTCTTGTT	-	E
7E1	TTTCACCTAGGGGCCAAAATAGTAGA	+	E
5I3	TCACACTGGCCTTTCCAGTTCTC	+	F
5B2	ATGGTTAATGTGAGCCACGCTGT	+	F
9B3	TTGCTCCTTTGTTCAGTTCATCAGA	+	F
9B6	TCTGCAACTGGAGACCCTGAAAC	+	F

8G2	TGGAACACAAAGGGAAAACACCA	+	F
8G1	ACGTGAAAGTGGGCATACAGCAT	-	F
28	AGCTGTGTGGTTCTCACTGGACA	+	F
5G5	CCCAGGGATGATCTAAGCTCTCG	-	F
7C10	TGTCCCTGGCACACTAAAGTCCT	-	F
9B7	CTTGTGGGAATCACAGTCCCTTG	-	F
5K8	CCTTCATATAACCCTGCAATCATGC	+	F
9C8	TGGCTCTTACAGGGAAAGCTCTG	+	F
37	ATACAAGCCCAAACACTGGCAGA	-	F
5L4	AGTGTCACTTTGCTTCCCGTCTG	+	F
5B3	CCGGGGTGATATTTACCAAGGTG	+	F
8E11	ACTGTTGGGGAGCAGCTCTTAGG	-	F
45	AAAAGAAGCCCCAAACATCTTGAA	-	F
8H4	AAACTGAGGCACAAAGGCATTGA	-	F
5F6	AAGCATGCATGGTCTGTGTCCTT	-	F
6E6	GGGGTTTGAAGACTGCTGGCTA	+	F
9E11	CTGTGGACCTGAAACCAATGGAC	+	G
9F12	CCTGGCATTCCATGGTTGATTC	-	G
9E7	GCTTGCAGAACTTAGCCTTGTGC	-	G
9H6	GATGCAGAGGTGTGTCCCTTCAG	+	G
31bis	CTGGGAATGGAGAGTTCACTTGC	-	G
10A3	CAAGGGACTGTGATTCCCACAAG	+	G
10B7	GCGCTGTTAGAGTTCAGGGACAC	+	G
10A2	GAACAAGGGTGCCGAGGTTAGAC	+	G
7G11	TGGCTGAGATTCCAAACTTGAGG	-	G
6E1	CGCCTTGATTTTCCATCAGACAG	-	G
9G6	AAGCCCTCCACAGGAAGAGTTTG	-	G
5I7	TCATTGACCTCTTTAGGCTTTCTTCA	-	G
6E8	AGGGGTCAGGTATCTGCTGCTTT	-	G
7C10	TGTCCCTGGCACACTAAAGTCCT	-	G
7E1	TTTCACCTAGGGGCCAAAATAGTAGA	+	G
8D5	TTCTAGCATGGCCTCAGGATGTG	-	G
8D4	GAGGTTGAAGGGTGCCTCTTGTT	-	G
8G2	TGGAACACAAAGGGAAAACACCA	+	G
8G1	ACGTGAAAGTGGGCATACAGCAT	-	G
45	AAAAGAAGCCCCAAACATCTTGAA	-	G
5L1	ATCCAGGTTAATGCGGGGACTT	-	H
55	ACCCACCTTCTTCTCTCCCCTTC	-	H
11c	CCCGCCTTCTAAATCCAGTTGAG	-	H
14'	GGAAATCCTGCCTAAGACAGTTGG	+	H
26	TCCTACTGCCTCAGCCTATCGTG	-	H
7'	GGCTTCCACGGTGTGCTCTATATC	+	H
9'	TGAGCTCTCCCATGAGGTTTCTG	-	H
25	GCCCCAGCTCCCAACATAAATAG	-	H
29	AGCCTAGGGCTCTGTTGGATGTC	-	H
31bis	ATACTTTTCGCCGGTGTCAGGAT	-	H
33	AGTTGCACCCTCAACTCCAAGAC	+	H
34	GCGCTGTTAGAGTTCAGGGACAC	+	H
38	GGGGCGTTCCCTAGACTAACAAA	-	H
53	CTGACACCTGCAGAAAGTGACCA	-	H
54	ATTAGGAGGTCAGGGGCATTTGA	-	H
6c	GTCATTGCAGACATGGTGGTGAC	+	H
30c	GGGCAGGACTTTTAATGGAGCAC	-	H
20d	CTGTCTCACGGTAGGGGACAGAC	-	H
5c	AAAGGGAAAGCTTGTGGGAACTG	+	H
42d	TGTTGTCACCTTAGATGGCTCTGG	-	H
