# Supplementary material for: A Systematic Enhancer Screen Using Lentivector Transgenesis Identifies Conserved and Non-Conserved Functional Elements at the Olig1 and Olig2 Locus
Source: PLoS One. 2010 Dec 29;5(12):e15741. doi: 10.1371/journal.pone.0015741 (PMC3012086; doi:10.1371/journal.pone.0015741)
Supplement: Table S3 — Summary of all injected clones. Coordinates, ID, Pool, integration status. (PDF) [file pone.0015741.s003.pdf]

Table S3

Start (Mm8)	End (Mm8)	Clone ID	Pool	Injected twice	Integrated
91052568	91056665	L7-356-P18.6	A		n
91085935	91088116	L4-356-P18.6	A		y
91090067	91092760	i06-356P18.5	A		y
91097068	91100177	F07-356P18.5	A		y
91106384	91108466	D7-356-P18.6	A		y
91109637	91111538	K1-356-P18.6	A		y
91113986	91116489	B2-356-P18.6	A		y
91119649	91122250	D02-356P18.5	A		y
91157209	91159549	A07-356P18.5	A		y
90992462	90995219	H08-356P18.5	B		y
90995220	90997339	I2-356P18.5	B		n
90999956	91001783	G01-356P18.5	B		y
91016502	91018759	F7-356-P18.6	B		y
91032548	91034365	C07-356P18.5	B		y
91049457	91052518	D06-356P18.5	B		y
91078438	91080339	D7-356P18.7	B		y
91096078	91097808	A5-356P18.5	B		y
91181242	91184052	i07-356P18.5	B	x	n
91184260	91188053	E6-356-P18.6	B	x	n
91009408	91012175	L01-356P18.5	C	x	n
91017932	91020851	B02-356P18.5	C	x	n
91116345	91119382	A3-356P18.5	C		y
91125129	91127152	D07-356P18.5	C		y
91127226	91130678	G5-356-P18.6	C		y
91130945	91132689	A01-356P18.5	C		y
91132624	91134636	F02-356P18.5	C		n
91138022	91140442	J6-356-P18.6	C		y
91153618	91156723	L04-356P18.5	C	x	y
91162463	91165047	F4-356-P18.6	C		n
91021023	91024244	L03-356P18.5	D		y
91024807	91027134	356P18.7.C5	D		y
91027385	91029416	E5-356-P18.6	D		y
91052759	91055215	J6-356P18.5	D		y
91056171	91059711	356P18.7E9	D		y
91071964	91073765	356.P18.8.B5	D		y
91082413	91085986	H04-356P18.5	D		y
91158569	91160533	L1-356-P18.6	D		y
91165080	91167632	B1-356-P18.6	D		y
91200560	91203773	L2-356-P18.5	D		y
91010795	91013098	B06-356P18.5	E		y
91023959	91026413	K4-356P18.5	E		y
91054035	91055972	E8-356-P18.6	E	x	n
91061492	91063760	A2-356P18.8	E		y
91166522	91168984	G06-356P18.5	E		y
91169486	91171384	C05-356P18.5	E		y
91171699	91174038	356.P18.8.B10	E		y
91176911	91178881	356P18.8D5	E	x	n
91186024	91189770	356P18.8D4	E	x	n
91198559	91201334	356P18.7E1	E	x	n
90995220	90997337	i03-356P18.5	F		y
91017932	91020851	B02-356P18.5	F	x	y
91036252	91038654	356.P18.9-B3	F		y
91040534	91042635	356.P18.9-B6	F		y
91060774	91062326	356.P18.8.G2	F	x	n
91074712	91076395	356.P18.8.G1	F	x	n

91095033	91096114	28	F		y
91114879	91116792	G05-356P18.5	F		y
91119296	91121563	356P18.7.C10	F	x	n
91123023	91125665	356.P18.9-B7	F		y
91132446	91134607	K8-356P18.5	F		y
91145978	91148056	356.P18.9-C8	F		y
91149913	91152319	37	F		y
91153618	91156723	L04-356P18.5	F	x	y
91155139	91157882	B03-356P18.5	F		y
91159456	91161383	356.P18.8.E11	F		y
91179971	91182599	45	F	x	n
91184260	91188053	E6-356-P18.6	F	x	n
91190549	91192157	356.P18.8.H4	F		y
91200259	91202576	F06-356P18.5	F		y
91013753	91014547	356P18.9E11	G		y
91034777	91038675	356P18.9-F12	G		y
91054035	91055972	E8-356-P18.6	G	x	y
91060774	91062326	356.P18.8.G2	G	x	n
91074712	91076395	356.P18.8.G1	G	x	n
91092940	91095282	356P18.9E7	G		y
91101872	91104576	356P18.9H6	G		n
91111221	91114051	31bis	G	x	y
91119296	91121563	356P18.7.C10	G	x	n
91120775	91123842	356P18.10-A3	G		y
91135691	91137979	356P18.10-B7	G		n
91137124	91139662	356P18.10-A2	G		n
91163066	91165057	356P18.7-G11	G		y
91163375	91166325	E1-356-P18.6	G		y
91168261	91170385	356P18.9G6	G		n
91176911	91178881	356P18.8D5	G	x	y
91181242	91184052	i07-356P18.5	G	x	y
91186024	91189770	356P18.8D4	G	x	y
91198559	91201334	356P18.7E1	G	x	y
91179971	91182599	45	G	x	n
91003792	91005208	6c	H		y
91005029	91006312	5c	H		y
91006178	91009107	7'	H		y
91009408	91012175	L01-356P18.5	H	x	y
91014243	91016402	9'	H		y
91028867	91031500	11c	H		y
91038522	91041473	14'	H		y
91065968	91068486	20d	H		y
91080093	91082418	25	H		y
91087971	91089883	26	H		y
91100121	91103069	29	H		y
91104717	91106432	30c	H		y
91111221	91114051	31bis	H	x	y
91134640	91136328	33	H		y
91136300	91137194	34	H		y
91152306	91153625	38	H		y
91189427	91191182	55	H		n
91192647	91195233	53	H		y
91195094	91197673	42d	H		y
91208048	91210312	54	H		y
