# Supplementary material for: A Systematic Enhancer Screen Using Lentivector Transgenesis Identifies Conserved and Non-Conserved Functional Elements at the Olig1 and Olig2 Locus
Source: PLoS One. 2010 Dec 29;5(12):e15741. doi: 10.1371/journal.pone.0015741 (PMC3012086; doi:10.1371/journal.pone.0015741)
Supplement: Table S4 — 3C primers and probe. (PDF) [file pone.0015741.s004.pdf]

Table S4

Relative to 5F7 (kb)	Forward primer (bait)	Reverse primer (target)	TaqMan probe (ligation site)
**-101.9**	GCATAGGCATGTTCTTTTTTCAAA	GGCTCCCAAATGTTTAGAATCACT	TCTTTAAACAGAAGCAGGCAGGTAGCT**GATC**CTA
**-22**	GCATAGGCATGTTCTTTTTTCAAA	CAGAGAGGTCAGGGTCAGTAAAGC	AGGCAGGTAGCT**GATC**CTCTGGTGTGT
**-15.1**	GCATAGGCATGTTCTTTTTTCAAA	AAGCCTTGCACTGATGAGTATCTG	AGAAGCAGGCAGGTAGCT**GATC**TGC
**1.1**	CAGCATAGGCATGTTCTTTTTTCA	CCTTCAGCCCTTGGCTTGA	TTTAAACAGAAGCAGGCAGGTAGCT**GATC**TAGTCC
**3.3**	TCTTTAAACAGAAGCAGGCAGGTA	TCAACAGAGGCAGCATGCAT	CT**GATC**CAGCCTTCATTCTCTGAA
**4.4**	AGCATAGGCATGTTCTTTTTTCAA	GCAGTTTATGGGCCATGTCA	CAGGCAGGTAGCT**GATC**TTCTCCTAG
**5.8**	TCTTTAAACAGAAGCAGGCAGGTA	GGCAACCAGGACCTACAACATC	CT**GATC**AGGATTCACCCTAGGCCAACTTACGTA
**9.7**	TCTTTAAACAGAAGCAGGCAGGTA	TGAATGGTGCAGCCCTACAC	CT**GATC**CACCCAATCCCCTTTAAC
**14.5**	TCTTTAAACAGAAGCAGGCAGGTA	CCCGAAGGAGGCATCTACAA	CT**GATC**AGAAAGGGCTCGGGCA
**35.1**	TCTTTAAACAGAAGCAGGCAGGTA	AGCCACACAAAGCCATCCAT	CT**GATC**ATGACAGTTAAGGATTCAAATAGA
**54.9**	GCATAGGCATGTTCTTTTTTCAAA	CTTCCCCTGGTGGTCTAATCTG	TAAACAGAAGCAGGCAGGTAGCT**GATC**TGACT
